# BMC Genetics reviewer acknowledgement 2014

**DOI:** 10.1186/s12863-015-0164-5

**Published:** 2015-03-25

**Authors:** Catherine J Potenski

**Affiliations:** BioMed Central, 233 Spring Street, New York, NY 10013-1578 United States of America

## Abstract

The editors of *BMC Genetics* would like to thank all our reviewers who have contributed to the journal in Volume 15 (2014).

**Khaled Abu-Amero**

Saudi Arabia

**Khaled Abu-Amero**

Qatar

**Jon Ahlinder**

Sweden

**Karen Aitken**

Australia

**Eduard Akhunov**

USA

**Emidio Albertini**

Italy

**Andreia Amaral**

Portugal

**Leif Andersson**

Sweden

**Toby Andrew**

UK

**Katja Anttila**

Finland

**Apostolos Apostolidis**

Greece

**Agustin Aranda**

Spain

**Sunil Arora**

India

**Juan Jose Arranz**

Spain

**Peter Askjaer**

Spain

**Giovanna Attene**

Italy

**Paul Auer**

USA

**Antonios Augustinos**

Greece

**David Aylor**

USA

**Yvonne Badke**

Belgium

**Emilia Bagnicka**

Poland

**Guihua Bai**

USA

**Fernando Sebastian Baldi Rey**

Brazil

**Gabriel Balmus**

UK

**Eliane Bandinelli**

Brazil

**Moinak Banerjee**

India

**Claiton Bau**

Brazil

**Ryan Baugh**

USA

**Esther Becker**

UK

**Dorothea Bedigian**

USA

**Piotr Bednarek**

Poland

**Daniele Bellavia**

Italy

**Bernhard Benkel**

Canada

**K.E. Bett**

Canada

**Mehul Bhakta**

USA

**Dwaipayan Bharadwaj**

India

**Mangesh Bhide**

Slovakia

**Jianmin Bian**

China

**Bill Black**

USA

**Paul Bloor**

Colombia

**Elizabeth Blue**

USA

**Lorenzo Bomba**

Italy

**Gouesnard Brigitte**

France

**Kevin Brix**

Canada

**Gudrun Brockmann**

Germany

**Karl Broman**

USA

**Richard Brown**

UK

**Anna Brüniche-Olsen**

Australia

**Sam Buckberry**

Australia

**Ana I. Burguete García**

Mexico

**Mark Burow**

USA

**Christopher Burtner**

USA

**Barbara Burwinkel**

Germany

**Bruno Buzatto**

Australia

**Diogo Cabral-De-Mello**

Brazil

**Mario Calus**

Netherlands

**Samuel Canizales**

Mexico

**Michele Caraglia**

Italy

**Ariel Castro**

Uruguay

**Stanislav Cepica**

Czech Republic

**Lisa Chakrabarti**

UK

**Alice H.D. Chan**

Singapore

**Giriraj Chandak**

India

**Bian Chao**

China

**Raquel Chaves**

Portugal

**Rui Chen**

USA

**Xiao-Yong Chen**

China

**Peng Cheng**

USA

**Li Cheng-Yun**

China

**Vikram Chhatre**

USA

**Yoon Shin Cho**

Korea, South

**Hon Yin Brian Chung**

Hong Kong

**Ki Wha Chung**

Korea, South

**Daniel Ciobanu**

USA

**Leigh Clark**

USA

**Veryan Codd**

UK

**Michael H. Crawford**

USA

**Richard Crooijmans**

Netherlands

**Yuehua Cui**

USA

**Kehui Cui**

China

**Dhanjit Kumar Das**

India

**Sonia Davila**

Singapore

**Roberta Davoli**

Italy

**Julie De Backer**

Belgium

**Thomas Debener**

Germany

**James Denvir**

USA

**Giovanni Destro Bisol**

Italy

**Sangeeta Dhaubhadel**

Canada

**Antonio Diaz**

Venezuela

**Roger Doyle**

Canada

**Tom Druet**

Belgium

**Qingzhang Du**

China

**Shiwei Duan**

China

**Naomi Duijvesteijn**

Netherlands

**Martina Durner**

USA

**Nduna Dzimiri**

Saudi Arabia

**Serge Edme**

USA

**Mohammed El Mzibri**

Morocco

**David Ellinghaus**

Germany

**Ronald Ellis**

USA

**Ahmed El-Sohemy**

Canada

**Mauricio Elzo**

USA

**Malena Erbe**

Germany

**Nir Eynon**

Australia

**Patricia Faivre Rampant**

France

**Md Fakruddin**

Bangladesh

**Bin Fan**

China

**Chao Fang**

USA

**Justin Faris**

USA

**Laura Fejerman**

USA

**Tao Feng**

USA

**Jesús Fernández**

Spain

**Luis Fernández**

Spain

**Rafael Fernández-Muñoz**

Spain

**Richard Finnell**

USA

**Lex Flagel**

USA

**Idhaliz Flores**

USA

**Laurence Flori**

France

**Jiri Forejt**

Czech Republic

**Marina Fortes**

Australia

**Glen Fox**

Australia

**Michael Francki**

Australia

**Elisabetta Frascaroli**

Italy

**Wenqing Fu**

USA

**Yong-Bi Fu**

Canada

**Silvia Fuselli**

Italy

**Agata Gadaleta**

Italy

**Sara Gandini**

Italy

**Guimin Gao**

USA

**Jonathan Gardner**

New Zealand

**Dorian Garrick**

USA

**Christiane Gebhardt**

Germany

**Nicolas Gengler**

Belgium

**Alessandra Gentile**

Italy

**Elena Gigli**

Italy

**Antonio Giordano**

USA

**Koumudi Godbole**

India

**Daniel Goedbloed**

Germany

**Shailendra Goel**

India

**Luis Gomez-Raya**

Spain

**Xun Gong**

China

**Louisa Goumidi**

France

**Manje Gowda**

India

**Eric Greer**

USA

**Christoph Grieder**

Switzerland

**Martien Groenen**

Netherlands

**Vince Kornél Grolmusz**

Hungary

**Sandeep Grover**

India

**Minxin Guan**

China

**Rudy Guerra**

USA

**Beatriz Gutiérrez-Gil**

UK

**Balazs Györffy**

Hungary

**Bianca Haase**

Australia

**Michael Hackenberg**

Spain

**Dominik Halas**

Canada

**Christopher Hamm**

USA

**Janetta Harbron**

South Africa

**Takeshi Hayashi**

Japan

**Tao He**

USA

**Qiuling He**

USA

**David Heckel**

Germany

**Laura Hernandez**

USA

**Alex Hewitt**

Australia

**Julie Hicks**

USA

**Paul Hocking**

UK

**David Holding**

USA

**Rikard Holmdahl**

Sweden

**Michael Vaclav Holmes**

UK

**Shigeru Honda**

Japan

**Firoz Hossain**

India

**Liping Hou**

USA

**Chris Hoze**

France

**Zhibin Hu**

China

**Zhiqiu Hu**

Canada

**Hailiang Huang**

USA

**Chun-Hsi Huang**

USA

**Celine Huber**

France

**Cornelis J. J. Huijsmans**

Netherlands

**Kent Hunter**

USA

**Julie Hussin**

Canada

**Eveline Ibeagha-Awemu**

Canada

**Akihiro Ikeda**

USA

**Ana Insua**

Spain

**Daniel Irimia**

USA

**Kristopher Irizarry**

USA

**Kamel Jabbari**

Germany

**Luc Janss**

Denmark

**Chatchawan Jantasuriyarat**

Thailand

**Bhavanath Jha**

India

**Baohu Ji**

USA

**Xueqiu Jian**

USA

**Bina Joe**

USA

**Michael Johnson**

Australia

**Haja Kadarmideen**

Denmark

**Thomas Källman**

Sweden

**Guolian Kang**

USA

**Henry J Kaplan**

USA

**Orla Keane**

Ireland

**Ephraim Kenigsberg**

Israel

**Hasan Khatib**

USA

**Pawan Khera**

USA

**Chiea Chuen Khor**

Singapore

**James Wraith Kijas**

Australia

**Joomyeong Kim**

USA

**Jun Kitano**

Japan

**Vinod Kurungara**

India

**Arthur Klatt**

USA

**Robert Klein**

USA

**Yann Klimentidis**

USA

**Chris Knight**

Denmark

**Sara Knott**

UK

**Michael Hans Kohn**

USA

**Sulev Koks**

Estonia

**Naoshi Kondo**

Japan

**Xiangyin Kong**

China

**Kon Konstantinov**

Australia

**Eszter Kotyuk**

USA

**Sheri Krams**

USA

**Juozas Kupcinskas**

Lithuania

**Bassam Lajin**

Syria

**Suman Lakhanpaul**

India

**Susan Lamont**

USA

**Stefano Landi**

Italy

**Taimour Langaee**

USA

**Janine Lasalle**

USA

**Hernan Laurentin**

Venezuela

**Fabienne Le Provost**

France

**Matthew Lebo**

USA

**Andres Legarra**

France

**Delphine Legrand**

France

**Sigrid Lehnert**

Australia

**Gabriel Leitner**

Israel

**Chun Li**

China

**Bingshan Li**

USA

**Chunying Li**

USA

**Gang Li**

USA

**Huawei Li**

China

**Hui Li**

China

**Huihui Li**

China

**Jingmei Li**

Singapore

**Ming Li**

China

**Yi Li**

Singapore

**Ming Li**

USA

**Rugang Li**

USA

**Zitong Li**

Finland

**Xu Lin**

China

**Huang Ling**

China

**Matt Littlejohn**

New Zealand

**Zhanjiang Liu**

USA

**Po-Ru Loh**

USA

**Jirong Long**

USA

**Luciana Lourenco**

Brazil

**Duc Lu**

Canada

**Tu Luan**

Norway

**Pierre Luisi**

Spain

**Jiangtao Luo**

USA

**Shiwen Luo**

China

**Guansheng Ma**

China

**Xiaochun Ma**

China

**Peipei Ma**

China

**Steven Mack**

USA

**Dylan Maddox**

USA

**Eva Madrid**

Spain

**Robert Maier**

Australia

**Christian Maltecca**

USA

**Licinio Manco**

Portugal

**Hideyuki Mannen**

Japan

**Yoshiro Mano**

Japan

**Balram Marathi**

Philippines

**Antonio Marco**

UK

**Gallegos-Arreola Martha Patricia**

Mexico

**Finian Martin**

Ireland

**Paula Martins-Lopes**

Portugal

**Kyonoshin Maruyama**

Japan

**Sarabjit Mastana**

UK

**Tara Mcdaneld**

USA

**Aline Meirhaeghe**

France

**Alessio Mengoni**

Italy

**Barbara Migeon**

USA

**Satoshi Mikawa**

Japan

**Makiko Mimura**

Japan

**Ignacy Misztal**

USA

**Andrew Mitchell**

Australia

**Toshiaki Mitsui**

Japan

**Paola Modesto**

Italy

**Battini Mohan Reddy**

India

**Grant Morahan**

Australia

**Takayuki Morisaki**

Japan

**Michael Mullen**

Ireland

**Yoshihiro Muneta**

Japan

**Zuzana Münzbergová**

Czech Republic

**Joram Mwacharo**

UK

**Xinzhi Ni**

USA

**Ezequiel Luis Nicolazzi**

Italy

**Motohide Nishio**

Japan

**Gloria Nombela**

Spain

**Dan Nonneman**

USA

**Evandro Novaes**

Brazil

**Gaetano Odierna**

Italy

**Bermseok Oh**

Korea, South

**Masafumi Ohtsubo**

Japan

**Morten Olesen**

Denmark

**Jose L. Oliver**

Spain

**Pablo Orozco-Terwengel**

UK

**Hakan Ozkan**

Turkey

**Malliya Gounder Palanichamy**

China

**Kai-Feng Pan**

China

**Manish Pandey**

India

**Josef Patzak**

Czech Republic

**Benedicte Paus**

Norway

**Hubert Pausch**

Germany

**Paulino Pérez**

Mexico

**Miguel Perez-Enciso**

Spain

**Andrea Cristina Peripato**

Brazil

**Hans-Peter Piepho**

Germany

**Francesc Piferrer**

Spain

**Elena Pilli**

Italy

**Pablo Pinedo**

USA

**Daniel Pinero**

Mexico

**Tommaso Pippucci**

Italy

**Eva Pisano**

Italy

**Odilia Popanda**

Germany

**Daniel Potaczek**

Germany

**Giorgio Prantera**

Italy

**Manoj Prasad**

India

**Nicolas Puillandre**

France

**Naveen Puppala**

USA

**Gao-Feng Qiu**

China

**Deyun Qiu**

Australia

**Petr Ráb**

Czech Republic

**Anthony Raizis**

New Zealand

**Satyawada Rama**

Rao India

**Harsh Raman**

Australia

**Mary Ramnitz**

USA

**Mojgan Rastegar**

Canada

**Alan Redd**

USA

**Giuseppe Reforgiato Recupero**

Italy

**Jun Ren**

China

**Jonathan Rios**

USA

**Robert Roberts**

Canada

**Luiz Rocha**

USA

**Dana Roldan**

Argentina

**Megan Rolf**

USA

**Dirk Roos**

Netherlands

**Guilherme Rosa**

USA

**Garry Rosewarne**

China

**Stephanie Rosse**

USA

**Yves Rosseel**

Belgium

**Anna Rita Rossi**

Italy

**María Susana Rossi**

Argentina

**Mahdi Saatchi**

USA

**Andrew Sandford**

Canada

**Daniele Santi**

Italy

**Adalberto Santos**

Brazil

**Shinji Sasazaki**

Japan

**Shunpei Sato**

Japan

**Arnold Saxton**

USA

**Massimo Scandura**

Italy

**Chris-Carolin Schoen**

Germany

**Holger Scholz**

Germany

**Martine Schroyen**

USA

**Heidi Schwarzenbach**

Germany

**Günther Schweizer**

Germany

**Federico Scossa**

Italy

**Stuart Scott**

USA

**Bertrand Servin**

France

**Qiuying Sha**

USA

**Xueyan Shan**

USA

**Igor Sharakhov**

USA

**Abhay Sharma**

India

**Min Shi**

USA

**Takeshi Shimogiri**

Japan

**Toshihiko Shiroishi**

Japan

**Moore Shoemaker**

USA

**Alicja Sieminska**

Poland

**Xueling Sim**

USA

**Domagoj Simic**

Croatia

**Kuldeep Singh**

India

**Andrew Singson**

USA

**Robert Smith**

Australia

**Alex Smith**

Canada

**Harold Smith**

USA

**Steve Smith**

Austria

**Timothy Smith**

USA

**Petr Smykal**

Czech Republic

**Warren Snelling**

USA

**Hidenobu Soejima**

Japan

**James Squires**

Canada

**Juan Steibel**

USA

**Don Stewart**

Canada

**Patrick Stover**

USA

**Ismo Stranden**

Finland

**Guang Sun**

Canada

**Lidan Sun**

China

**Xiangqing Sun**

USA

**Akiko Takasuga**

Japan

**Nicholas Tan**

USA

**Zheng-Zheng Tang**

USA

**Jeremy Taylor**

USA

**Chunfa Tong**

China

**Sissades Tongsima**

Thailand

**Shaun Townsend**

USA

**Roberto Tuberosa**

Italy

**Meryanne Tumonggor**

USA

**Takayuki Ueno**

Japan

**Guilherme T Valente**

Brazil

**Johannes Bchm Van Kaam**

Italy

**Mannis Van Oven**

Netherlands

**Annalisa Varriale**

Italy

**Monica Vetter**

USA

**Johanna Vilkki**

Finland

**Teresa Villarreal-Molina**

Mexico

**John Vincent**

Canada

**Jeane Visentainer**

Brazil

**Marco Volante**

Italy

**John Vontas**

Greece

**Shigeharu Wakana**

Japan

**Ronald Walter**

USA

**Chunkao Wang**

USA

**Chenguang Wang**

USA

**Haijian Wang**

China

**Jin-Jun Wang**

China

**Ming Li Wang**

USA

**Wen Wang**

USA

**Zhong Wang**

USA

**Xingjun Wang**

China

**Yaqun Wang**

USA

**Zuoheng Wang**

USA

**Jonas Warringer**

Sweden

**Toshio Watanabe**

Japan

**Dirk Wedekind**

Germany

**Changshuai Wei**

USA

**Peng Wei**

USA

**Wen-Hua Wei**

UK

**Olga Wellnitz**

Switzerland

**Yangjun Wen**

China

**Roman Wenne**

Poland

**Jon White**

UK

**Klaus Wimmers**

Germany

**Naomi Wray**

Australia

**Chih-Chieh Wu**

Taiwan

**Song Wu**

USA

**Weiren Wu**

China

**Xiaowei Wu**

USA

**Fangming Xie**

Philippines

**Conghua Xie**

China

**Chao Xing**

USA

**Yongzhong Xing**

China

**Hongyan Xu**

USA

**Jianlong Xu**

China

**Shuhua Xu**

China

**Yaji Xu**

USA

**Chao-Yie Yang**

USA

**Jie Yang**

USA

**Jianzhong Yang**

China

**Rong-Cai Yang**

Canada

**Liming Yang**

USA

**Wenyu Yang**

China

**Guoyou Ye**

Philippines

**Guoyou Ye**

Australia

**Kyung Lim Yoon**

Korea, South

**Bing Yu**

USA

**Jianming Yu**

USA

**Lixing Yuan**

China

**Gen Hua Yue**

Singapore

**Junming Yue**

USA

**Mohamed Zaghloul**

Egypt

**Robert Zemetra**

USA

**Weiwei Zhai**

Singapore

**Haimao Zhan**

USA

**Ben Zhang**

China

**Deqiang Zhang**

China

**Fengyu Zhang**

China

**Yuan-Ming Zhang**

China

**Xiquan Zhang**

China

**Wei Zhao**

USA

**Yonglan Zheng**

USA

**Yang Zhiling**

China

**Igor Zhimulev**

Russian Federation

**Wei Zhong**

China

**Chengsong Zhu**

USA

